# 

**Published:** 1852-10

**Authors:** 


### CONTENTS.

### ORIGINAL COMMUNICATIONS.

Page
Proceedings of the Mississppi Valley Association of Dental Surgeons,.... 1
Dr. Taft's Address before the Mississppi Valley Ass'n of " " .... 6
Springing of Plates. By Dr. J. Taylor,.......................... 10

### SELECTIONS.

A New Method of supplying Artificial Teeth and Gums. By Wm. M. Hunter, Dentist,.......................... 14
Report of Annual Meeting of the American Society of Dental Surgeons, . 25
Gross on Excision of the Maxillary Bone,.......................... 30
Remarks on Making Gold Plate and Solder. By B. Wood, M. D.,....... 47
Chase on Making Metallic Casts, &c.......................... 50

### CORRESPONDENCE.

Letter from Dr. J. Murray,.......................... 53

### EDITORIAL.

Mississippi Valley Association of Dental Surgeons,".......... 55
Ohio Dental College Association,.......................... 55
Pittsburgh Dentists,.......................... 56
Am. Society of Dental Surgeons,.......................... 56
Making Gold Plate and Solder,.......................... 56
A New Mode of Treating Exposed Dental Nerves,.......................... 57
The Register.......................... 57
To the Profession,.......................... 57
Advertising,.......................... 58
Arrearages for the Register,.......................... 58
Agents, .......................... 58

